# Maternal health literacy status and associated factors among pregnant women: a cross-sectional study in Chongqing, China

**DOI:** 10.3389/fpubh.2026.1824347

**Published:** 2026-07-16

**Authors:** Hong Yang, Yi Yang, Ping Huang, Guihua Huang, Haiyan Li, Jing Tan, Xiaoyan Xu, Dan Yu, Yujiao Wu, Ling Zhang, Li Ren

**Affiliations:** 1 Department of Obstetrics and Gynecology, Women and Children's Hospital of Chongqing Medical University, Chongqing, China; 2 Department of Obstetrics and Gynecology, Chongqing Health Center for Women and Children, Chongqing, China; 3 Department of Health Education, School of Military Preventive Medicine, Army Medical University (Third Military Medical University), Chongqing, China

**Keywords:** health literacy, associated factors, maternal health literacy, pregnancy, pregnant women

## Abstract

**Background:**

Maternal health literacy (MHL) plays a critical role in improving perinatal outcomes. This study aimed to investigate MHL status and its associated factors among pregnant women in Chongqing, China.

**Methods:**

This multi-center cross-sectional study included 1025 low-risk pregnant women between September 1 and December 31,2023, at 6 hospitals in Chongqing, and the participants were enrolled with equal distribution across trimesters (1:1:1). Data were collected using medical records, sociodemographic questionnaire and MHL questionnaire. Logistic regression was used to examine factors associated with MHL.

**Results:**

The adequate MHL rate was 7.9% among pregnant women in this study, with a higher rate (9.66%) in the dimension of basic knowledge. However, there was no significant difference in the three subscales of MHL (*P* = 0.070). Furthermore, there was no significant difference in the rate of MHL across different trimesters (*P* = 0.058), although the rate was higher in the first trimester (9.94%) and lower in the second trimester (5.19%). Higher educational level (OR = 2.584, 95% CI = 1.045–6.391), worries about the effect of pregnancy on work (OR = 2.287, 95% CI = 1.386–3.772), and attendance at pregnancy school (OR = 2.917, 95% CI = 1.434–5.934) were positively associated with the rate of MHL. Participants from rural areas (OR = 0.365, 95% CI = 0.218–0.610), ethnic minorities (OR = 0.297, 95% CI = 0.103–0.859), and abnormal results of prenatal screening (OR = 0.259, 95% CI = 0.078–0.861) were negatively associated with the rate of MHL.

**Conclusions:**

The adequate MHL rate in Chongqing was relatively low, and it was positively associated with higher educational level, worries about the effect of pregnancy on work, and attendance at pregnancy school, but was negatively associated with living in rural areas, ethnic minorities, and abnormal results of prenatal screening. Based on this evidence, future research could improve the rate of MHL.

## 1 Introduction

Maternal and child health is a critical indicator of societal development and national public health standards. Despite advancements in medical technology, preventable maternal and neonatal mortality, along with other adverse pregnancy outcomes, remain persistent global challenges (1–3). However, relying on healthcare services alone is insufficient to address these tissues. Individual health literacy has thus emerged as a crucial and modifiable determinant of health outcomes (4).The World Health Organization (5) defines health literacy (HL) as “the cognitive and social skills that determine an individual's motivation and ability to access, understand, and use information to promote and maintain health.” Renkert and Nutbeam (6) integrated HL with maternal characteristics to the concept of maternal health literacy (MHL), which is applicable to pregnant women and postpartum women. MHL is defined as the ability of pregnant women to access, understand, and apply health information for promoting maternal and child health, including three dimensions: basic knowledge, healthy lifestyle and behavior, and basic skills. Research has indicated that rate of MHL strongly associated with health outcomes for both mothers and their children (7).This is achieved through the promotion of beneficial health behaviors. Specifically, adequate MHL rate empower women to proactively seek medical information, adopt healthier lifestyles, and adhere to essential care practices such as regular antenatal care (8) and appropriate use of supplements (9). Furthermore, it supports positive perinatal and postnatal choices, such as actively pursuing vaginal delivery when feasible (10), adhering to breastfeeding practices (11), improving childhood vaccination compliance (12), all of which contribute to better health and potentially lower healthcare costs.

MHL has gained increasing international attention. However, most empirical studies have been conducted in high-income developed countries (5, 13), such as those in North America and Europe. While these studies have provided a crucial foundation for theoretical frameworks, measurement tools, and established associations with health outcomes, their findings require further empirical validation in non-Western and developing regions—particularly those with distinct social structures and traditional culture, such as China. China is undergoing rapid socioeconomic transformation. Its healthcare system, demographic structure, and cultural practices, including its large migrant population and tradition of intergenerational childcare (14–16), differ significantly from those in high-income countries. This may limit the direct applicability of foreign research and intervention models.

The existing domestic studies often focused on single dimensions of MHL (17, 18), such as basic knowledge about pregnancy and childcare, or only health-related behaviors, or only functional skills like reading ability. Although these single-dimension approaches are easy to administer, they may oversimplify the construct of MHL, which is recognized as a multidimensional concept. Consequently, few studies have simultaneously examined all three dimensions in a single sample, limiting our understanding of how these components interrelate and which dimension is most deficient in specific populations.

The present study employs a multidimensional MHL questionnaire developed by the China Maternal and Child Health Care Association (19), which includes three subscales: basic knowledge, healthy lifestyle and behaviors, and basic skills. This instrument has been used in several previous studies conducted in other developed Chinese cities, such as Beijing (20) and Shanghai (21). These developed regions have stronger economies, more established healthcare systems, and generally higher rate of health literacy. In contrast, far less attention has been paid to central and western regions, which are often characterized by a distinct urban–rural duality, uneven economic development, and large concentrations of ethnic minority populations. Moreover, existing research using this instrument has not systematically explored the differences across all three trimesters of pregnancy. Therefore, knowledge gaps remain regarding (1) the current adequate MHL rate among pregnant women in less-developed western Chinese regions; (2) whether the three dimensions of MHL differ significantly within the same population; and (3) which factors independently predict adequate MHL.

Identifying factors associated with MHL is crucial for several reasons. First, such knowledge can help healthcare providers and policymakers target health education resources to subgroups with the greatest needs. Second, understanding modifiable factors can guide the design of feasible and effective interventions to improve MHL. Third, exploring non-modifiable but identifiable risk factors enables early identification of populations who may require additional support. Without this evidence, interventions may be delivered in a uniform manner that fails to address specific barriers faced by different groups of pregnant women.

Chongqing, as the only direct-administered municipality in western China, presents a unique socio-geographic profile that integrates a major metropolis with vast rural and mountainous areas. Chongqing had a resident population of approximately 31.87 million in 2025, with a birth rate of 4.64% and a natural population growth rate of −4.61% (22). In 2024, the perinatal mortality rate, infant mortality rate and under-five mortality rate were 0.99%, 1.92%, and 2.87%, respectively (23). A 2016 survey conducted in Beibei District of Chongqing reported adequate MHL rate only 1.79% among pregnant women, further supporting the relatively low rate in this region (24). As such, the challenges faced by pregnant women in accessing health information and utilizing healthcare services in Chongqing are both distinctive and representative of many less-developed regions.

Therefore, this study aimed to address the following research questions: (1) What is the current adequate MHL rate among pregnant women in Chongqing, China? (2) What sociodemographic and obstetric factors are independently associated with MHL rate among this population? For healthcare providers in low- and middle-income countries facing similar health inequities, these findings may serve as an evidence base for developing interventions to improve MHL in pregnant women.

## 2 Materials and methods

### 2.1 Sampling procedure

Stratified sampling was performed. According to the *Chongqing Health Statistics Yearbook 2022*, Chongqing is divided into one metropolitan area and two urban agglomerations, covering its major geographical and population distribution. Sample size was calculated using a single proportion formula: *n* = *Z*^2^×*P*×*(1-P)/d*^2^, where *Z* = 1.96 (95% confidence), the estimated proportion of pregnant women with adequate MHL was set at *P* = 0.15 based on prior regional data, and allowable error *d* = 0.025, yielding a minimum of 784. Accounting for a 15% non-response rate, the target was 900–1,050 participants.

To ensure the representativeness of the sample and to cover institutions with different levels of medical resources, in the first stage, sampling was conducted proportionally according to the number of live births in each region of Chongqing in 2022, while also considering regional coverage, the distribution of medical resources, obstetric service capacity, and the feasibility of field implementation. In 2022, the total number of live births in Chongqing was 183,875. The numbers of live births in the Main Metropolitan Area, the Northeast Chongqing Three Gorges Reservoir Area Urban Agglomeration, and the Southeast Chongqing Wuling Mountain Area Urban Agglomeration were 115,364, 45,956, and 22,555, respectively, accounting for 62.74%, 24.99%, and 12.27% of all live births. Because the Main Metropolitan Area had the highest proportion of live births, the greatest demand for maternal health services, and relatively concentrated medical resources, three hospitals were randomly selected from this area. Because the Northeast Chongqing Three Gorges Reservoir Area Urban Agglomeration had an intermediate number of live births and covered a relatively large geographic area, two districts/counties, Zhong County and Yunyang County, were selected from northeastern Chongqing. Because the Southeast Chongqing Wuling Mountain Area Urban Agglomeration had a relatively small number of live births, one district/county, Xiushan Tujia and Miao Autonomous County, was selected from southeastern Chongqing. In the selected districts/counties, maternal and child health hospitals or general hospitals were chosen as study sites.

In the second stage, convenience sampling was conducted in maternal and child health hospitals or general hospitals in the selected areas, at least 150 questionnaires distributed at each site. The ratio of samples in the first, second, and third trimesters was set at 1:1:1.Because field recruitment was affected by antenatal clinic volume, the study period, institutional cooperation, and the number of eligible pregnant women attending the selected hospitals, the final number of valid participants was not completely consistent with the initially planned sample allocation based on the proportion of live births. Finally, a total of 1,025 pregnant women participated in this study, including 447 from metropolitan area and 578 from two urban agglomerations.

### 2.2 Participants

This multi-center cross-sectional study collected data between September 1 and December 31, 2023. Women who met the following inclusion criteria were invited to the study: diagnosed with a single pregnancy, age ≥ 18 years old, having basic reading and comprehension skills. Participants were excluded for any one of the following reasons: had severe pregnancy-related comorbidities or complications, such as hypertensive disorders of pregnancy; or had communication impairments or mental disorders.

### 2.3 Questionnaire

Data were obtained through face-to-face interviews using the two instruments in this study. The first instrument was a sociodemographic questionnaire developed by the researchers, with a total of 22 items, covering demographic characteristics such as participants' age, educational level, employment status, monthly income, and obstetric characteristics such as obstetric history and complications.

The second instrument was a maternal and infant health literacy (MHL) questionnaire, developed and compiled by the China Maternal and Child Health Care Association based on Article 55 of *Basic Knowledge and Skills of Maternal and Child Health Literacy* (19). The questionnaire comprises 35 items covering key health topics, including nutrition and physical activity during pregnancy, breastfeeding practices, neonatal care, and other essential maternal and child health issues (Supplementary Data sheet 1). It consists of three dimensions: basic knowledge (22 items), healthy lifestyle and behaviors (8 items), and basic skills (5 items). Each item has five response options, with only one correct answer, a correct answer is assigned 1 point, whereas an incorrect answer is assigned 0 points. The total score is calculated by summing the scores for all items. Based on the monitoring of health literacy among Chinese residents under the national “Health Literacy Promotion Action Program,” Participants who achieved 80% or more of the total score (≥80) were considered to have adequate MHL, this assessment criterion is widely adopted in domestic surveys on MHL (25–27).The questionnaire had high validity and reliability, with a Cronbach's alpha coefficient of 0.897 calculated based on the data from the 1,025 participants.

### 2.4 Data collection

Data were collected by trained research nurses affiliated with the participating hospitals. Before data collection, all research staff received standardized training on questionnaire administration and participant communication. Questionnaires were administered in a self-report format during participants' scheduled antenatal clinic visits. For participants with limited reading ability, the questions were read aloud by a research nurse without providing directional cues. The average time required to complete both questionnaires was approximately 15–20 min. Participants' responses were anonymous, and all data were stored securely.

### 2.5 Data analysis

All data were analyzed using IBM SPSS Statistics for Windows, Version 26.0 (IBM Corp., Armonk, NY, USA). Sociodemographic and obstetric data were described as mean ± standard deviation (SD) and frequency [percentage *n* (%)], respectively. Chi-square (χ^2^) test analysis was used to evaluate the differences in the subgroups. After adjusting for Sociodemographic and obstetric data and related variables with *P* < 0.05 in the primary analysis, data was entered into a logistic regression analysis to investigate the association between demographic and obstetric factors on MHL. Statistical significance was set at *P* < 0.05 (two-sided). This cross-sectional study was reported in accordance with the STROBE guidelines. The completed STROBE checklist is provided as Supplementary Data Sheet 2.

### 2.6 Ethical considerations

This study was approved by the Ethics Committee of the Chongqing Health Center for Women and Children (NO: 2024-041). Prior to the study, all participants were informed of the study objectives, procedures, potential risks, and benefits, and provided written informed consent. Participant privacy was strictly protected, and all data were kept confidential. Participants were informed of their right to withdraw at any time without any negative consequences.

## 3 Results

A total of 1,025 women, 447 from metropolitan area and 578 from two urban agglomerations, participated in this study. The mean age of the participants was 29.39 ± 4.17 years old, and most (86.65%) were < 35 years old. Of these, 97.03% were married, and over 80% of the pregnant women and their husbands had a high school degree or above. Regarding obstetric characteristics, approximately half of the women were multiparous (47.25%), and only 25% had an abnormal pregnancy history. Most women (67.58%) had never attended pregnancy schools, 23.41% had participated once or twice during pregnancy, and only 9% had attended more than three times. The participants' characteristics are shown in Table 1.

**Table 1:** Sociodemographic and obstetric characteristic of participants.

Variables	Option	Inadequate MHL		Adequate MHL		Total		χ2	*P*-value
		*N* (%)		*N* (%)		*N* (%)			
Age	< 35	818	86.65	77	95.06	895	87.32	4.763	0.029
	≥35	126	13.35	4	4.94	130	12.68		
Ethnicity	Han	797	84.43	77	95.06	874	85.27	–	0.008
	Ethnic minorities	147	15.57	4	4.94	151	14.73		
Marriage	Single	23	2.44	3	3.70	26	2.54	–	0.306
	Married	916	97.03	77	95.06	993	96.88		
	Divorced	5	0.53	1	1.23	6	0.58		
living environment	Urban areas	393	41.63	54	66.67	447	43.61	19.013	0.000
	Rural areas	551	58.37	27	33.33	578	56.39		
Participants' education level	Middle school or below	153	16.21	6	7.41	159	15.51	4.408	0.036
	High school or above	791	83.79	75	92.59	866	84.49		
Husbands' education level	Middle school or below	162	17.16	7	8.64	169	16.49	3.932	0.047
	High school or above	782	82.84	74	91.36	856	83.51		
Monthly income (CNY)	< 5,000	212	22.46	15	18.52	227	22.15	11.189	0.011
	5,000~	308	32.63	41	50.62	349	34.05		
	8,000~	283	29.98	18	22.22	301	29.37		
	15,000~	141	14.94	7	8.64	148	14.44		
Participants' occupation	Unemployed/housewives	285	30.19	19	23.46	304	29.66	1.621	0.203
	Employed	659	69.81	62	76.54	721	70.34		
Husbands' occupation	Unemployed	38	4.03	2	2.47	40	3.90	–	0.764
	Employed	906	95.97	79	97.53	985	96.10		
Health insurance	No	262	27.75	18	22.22	280	27.32	–	0.348
	Yes	682	72.25	63	77.77	745	72.68		
Gestational weeks	The first trimester	299	31.67	33	40.74	332	32.39	5.689	0.058
	The second trimester	329	34.85	18	22.22	347	33.85		
	The third trimester	316	33.47	30	37.04	346	33.76		
Parity	Primigravida	498	52.75	46	56.79	544	53.07	0.488	0.485
	Multipara	446	47.25	35	43.21	481	46.93		
Pregnancy condition	Wanted	621	65.78	58	71.60	679	66.24	1.130	0.288
	Unwanted	323	34.22	23	28.40	346	33.76		
Abnormal pregnancy history	No	708	75.00	71	87.65	779	76.00	6.549	0.010
	Yes	236	25.00	10	12.35	246	24.00		
Prenatal screening	No	450	47.67	35	43.21	485	47.32	0.595	0.440
	Yes	494	52.33	46	56.79	540	52.68		
Results of prenatal screening	Normal	811	85.91	78	96.30	889	86.73	–	0.006
	Abnormal	133	14.09	3	3.70	136	13.27		
Anemia	No	783	82.94	68	83.95	851	83.02	0.154	0.817
	Yes	161	17.06	13	16.05	152	14.83		
Gain weight (Kg)	< 12	782	82.84	69	85.19	851	83.02	0.291	0.589
	≥12	162	17.16	12	14.81	174	16.98		
Worries about the effect of pregnancy on work	No	669	70.87	42	51.85	711	69.37	12.696	0.000
	Yes	275	29.13	39	48.15	314	30.63		
Attendance at pregnancy school	Never	638	67.58	35	43.21	673	65.66	21.154	0.000
	1 2 times	221	23.41	30	37.04	251	24.49		
	≥3 times	85	9.00	16	19.75	101	9.85		
Sleeping status	Terrible	279	29.56	14	17.28	293	28.59	5.503	0.019
	Good	665	70.44	67	82.72	732	71.41		
Self-evaluated health status	Good	681	72.14	70	86.42	751	73.27	–	0.008
	Middle	253	26.80	10	12.35	263	25.66		
	Terrible	10	1.06	1	1.23	11	1.07		

*N* (%) in this column indicates the frequency and percentage of the data; MHL, Maternal health literacy; Participants who achieved 80% or more of the total score (≥80) were considered to have adequate MHL.

Adequate MHL rate among pregnant women was 7.90% in this study, with a higher rate (9.66%) in the dimension of basic knowledge, but there was no significant difference in the three subscales of MHL (*P* = 0.070). And there was no significant difference in the adequate MHL rate in different trimesters (*P* = 0.058), but adequate MHL rate were higher among participants in the first trimester (9.94%) and lower among those in the second trimester (5.19%) (Table 2).

**Table 2:** Adequate MHL rate among pregnant women.

Variables	Adequate MHL (*N* %)				χ2	*P*-value
	Total	First trimester	Second trimester	Third trimester		
MHL	81(7.90)	33(9.94)	18(5.19)	30(8.67)	5.689	0.058
Basic knowledge	99(9.66)	45(13.55)	50(14.41)	4(1.16)	–	0.000
Healthy lifestyle and behavior	76(7.41)	29(8.73)	15(4.32)	32(9.25)	7.370	0.024
Basic skills	73(7.12)	36(10.84)	15(4.32)	22(6.36)	11.366	0.003
χ2	5.325	3.945	33.174	–		
*P*-value	0.070	0.150	0.000	0.000		

*N* (%) in this column indicates the frequency and percentage of the data; MHL, Maternal health literacy.

Table 1 summarizes the participants' age, ethnicity, living environment, participants and their husbands' educational level, monthly income, abnormal pregnancy history, results of prenatal screening, worries about the effect of pregnancy on work, attendance at pregnancy school, sleeping status and self-evaluated health status which showed significant associations with the rate of MHL using the χ^2^ test (all *P* < 0.05).

And the variance inflation factors (VIFs) range from 1.005 to 1.015, indicating no multicollinearity among the variables.

Variables included in the logistic regression model were selected based on a combination of χ^2^ test results (*P* < 0.05), evidence from previous literature and clinically important variables, such as gestational weeks and parity, were considered as potential confounders and were evaluated in the model regardless of their statistical significance in χ^2^ test. The Hosmer-Lemeshow goodness-of-fit test yielded a chi-square value of 5.1 (df = 6, *P* = 0.531), indicating that the logistic regression model fitted the data adequately. The discriminative ability of the model was evaluated using the receiver operating characteristic (ROC) curve. The area under the curve (AUC) was 0.732(95% CI = 0.68–0.78), suggesting moderate discriminative ability for distinguishing between pregnant women with adequate vs. inadequate MHL. As shown in Table 3, the rate of MHL was positively associated with participants' higher educational level (OR = 2.584, 95% CI = 1.045–6.391), worries about the effect of pregnancy on work (OR = 2.287, 95% CI = 1.386–3.772), attendance at pregnancy school (OR = 2.917, 95% CI = 1.434–5.934). However, participants from rural areas (OR = 0.365, 95% CI = 0.218–0.610), ethnic minorities (OR = 0.297, 95% CI = 0.103–0.859), and abnormal results of prenatal screening (OR = 0.259, 95% CI = 0.078–0.861) had lower odds of adequate MHL.

**Table 3:** Logistic regression analysis of factors associated with MHL.

Variables	*P*	OR	95% C.I. for EXP (B)	
			Lower	Upper
Living environment				
Rural areas	0.000	0.365	0.218	0.610
Reference: urban areas				
Ethnicity				
Ethnic minorities	0.025	0.297	0.103	0.859
Reference: Han				
Participants' education level				
High school or above	0.04	2.584	1.045	6.391
Reference: Middle school or below				
Worries about the effect of pregnancy on work				
Yes	0.001	2.287	1.386	3.772
Reference: No				
Attendance at pregnancy school				
1 2 times	0.001	2.464	1.421	4.272
Reference: Never				
≥3 times	0.003	2.917	1.434	5.934
Reference: Never				
Results of prenatal screening				
Yes	0.028	0.259	0.078	0.861
Reference: No				

MHL, Maternal health literacy.

## 4 Discussion

### 4.1 Low rate of MHL among pregnant women in Chongqing

In this study, the proportion of pregnant women with adequate MHL was low, which was not only lower than rates reported in coastal developed regions of China (28) but also below the range reported in most high-income countries (29). This disparity may be attributed to several factors. Chongqing, has substantial urban—rural disparities and relatively underdeveloped healthcare infrastructure in rural and mountainous areas. In this study, approximately half of the participants were from county-level areas, which may have contributed to the lower rate of MHL. The low rate of MHL was not an isolated phenomenon but rather reflected the broader challenges associated with the dissemination of maternal health knowledge within a specific sociocultural context. This reminds us that current maternal health education may be substantially inadequate in terms of both coverage and effectiveness.

In this sample, MHL rates were higher among participants in the first and third trimesters than those in the second trimester. However, because of our cross-sectional design, these differences should not be interpreted as within-person changes. Higher MHL rates in the first trimester may reflect increased anxiety and a heightened motivation to actively seek information following pregnancy confirmation (30). The lower MHL rates observed in the second trimester may be associated with the relative stabilization of physical and mental health following the resolution of early pregnancy discomforts, leading to reduced self-care awareness. Additionally, as the second trimester is the longest stage of gestation, and given that the majority of pregnant women in this study had normal results throughout prenatal examinations, they may tend to neglect learning maternal and infant health knowledge or even engage in selective information avoidance (31). The higher MHL rates during the third trimester may reflect that impending childbirth motivates pregnant women to re-engage with healthcare providers and actively seek information regarding labor and newborn care (32). This result differs from a previous study (33) suggesting that MHL rates increase with pregnancy progression and knowledge accumulation. However, these differences do not necessarily imply intra-individual changes over the course of pregnancy. Nevertheless, these findings suggest that healthcare professionals should consider developing phased and targeted health education programs, with particular emphasis on enhancing the frequency of proactive health education during the second trimester. Optimizing the content and design of educational materials may further contribute to improvements in maternal health literacy.

Regarding the three MHL subscales, the proportion of participants with adequate MHL was descriptively higher for basic knowledge than for behaviors and skills, although pairwise comparisons showed no statistically significant differences. One possible interpretation is that acquiring health knowledge does not automatically lead to corresponding health behaviors or skills—a phenomenon often discussed in the Knowledge-Attitude-Practice (KAP) model (34). However, the current cross-sectional design did not directly measure the sequence or causal pathway from knowledge to behavior. Therefore, this interpretation remains speculative and requires empirical testing in longitudinal studies. Nevertheless, based on existing literature (35, 36), interactive and experiential methods, such as scenario simulations, role-playing, and peer demonstrations, have been suggested to support health behavior change. These approaches may be considered in future interventions aimed at improving MHL, though their effectiveness specifically in this population needs further evaluation.

### 4.2 Factors associated with MHL among pregnant women

In the following discussion, to enhance conceptual clarity, we refer to these three core actions: accessing, understanding, and using health information, as the key components of MHL (6).

This study found several factors were positively associated with adequate MHL. First, higher educational level was positively associated with the rate of MHL, consistent with prior research (31). Higher education may correspond to greater cognitive skills that facilitate accessing written prenatal materials, understanding medical recommendations, and using information for health decisions. However, due to the cross-sectional design, the association merely indicates that women with higher education were more likely to have adequate MHL in this sample. Second, worries about the effect of pregnancy on work were also positively associated with MHL. From the theoretical perspective, expressing concern about work-related challenges during pregnancy may reflect an awareness of potential health risks. This awareness itself may require the ability to access relevant information, to understand the implications of those risks for maternal and fetal health, and to use that information for health-protective decisions (29, 37). Both may be part of a broader orientation toward actively engaging with health information during pregnancy. Conversely, women who lack the skills to access, understand, or use health information may also be less likely to recognize work-related risks. They may report less worry-not because they are less exposed, but because their lower MHL limits their awareness (31).In our study, the association could reflect that higher MHL is associated with greater identification of work-related concerns, or that expressing worry is associated with seeking health information, or that both are influenced by unmeasured factors such as occupational category, workplace support, or job security. Third, attending pregnancy school showed a positive association with the rate of MHL. Pregnancy schools are structured settings where women can practice accessing prenatal knowledge, understanding risk and self-care recommendations, and using information for behaviors such as recognizing danger signs or preparing for delivery (38). However, over two-thirds of our participants had never attended one and previous research has noted format monotony and lack of practical content (39). Nevertheless, our study cannot determine whether attending pregnancy school leads to higher MHL or whether women with higher MHL are more likely to attend pregnancy school (or both). The association merely indicates that the two variables are related in this population.

This study also found that participants from rural areas, ethnic minorities, and abnormal results of prenatal screening were negatively associated with MHL. Pregnant women in rural areas had lower MHL rates than those in urban areass, which is consistent with previous descriptive findings (40, 41). From the theoretical perspective, rural environments may offer fewer resources for accessing evidence-based maternal health information, greater challenges in understanding clinical recommendations due to limited health literacy support, and less opportunity to use information for daily self-care decisions. In addition, insufficient local emphasis on maternal and child health initiatives in some township regions may also be associated with this observed gap (42). However, because of the cross-sectional design, the association simply indicates that rural living and lower MHL co-occur in this population.

Our results showed that ethnic minority pregnant women had significantly lower MHL, consistent with previous findings (43, 44).This disparity may be explained by the fact that ethnic minority populations are disproportionately concentrated in remote, medically underserved areas. These findings imply that healthcare providers should move beyond generic health education and instead offer culturally tailored guidance that addresses the specific needs of ethnic minority women. Practical strategies could include providing translated or multilingual materials, training community health workers from similar ethnic backgrounds, and integrating culturally appropriate content into routine prenatal education, thereby improving their MHL and promoting greater health equity.

Abnormal prenatal screening results were also negatively associated with adequate MHL. Conditions such as gestational diabetes (45) and hypertension (46) were associated with unhealthy behaviors in pregnant women, reflecting inadequate MHL. On the one hand, pregnant women with low MHL often face difficulties accessing and understanding prenatal health information, leaving them unable to effectively prevent avoidable health risks. On the other hand, when confronted with abnormal test results, their adherence to medical advice is relatively poor, which may further exacerbate adverse pregnancy outcomes. It is equally important to promptly identify MHL-related support needs when pregnant women receive such abnormal results. Providing clear, actionable information and continuous follow-up guidance may be more effective than conventional health education in empowering them to manage their own health.

### 4.3 Limitations and future research directions

This study is the first to investigate the rate of maternal health literacy and influencing factors in pregnant women in Chongqing, China, and the results reflect the position in Chongqing. However, this study had several limitations. First, this study did not measure potentially relevant factors such as mental health status and social support. Future research should consider including these variables to explore their possible association with MHL. Second, owing to its cross-sectional design, it captures MHL rates at a single time point and can only describe differences between groups of pregnant women at different trimesters. It cannot assess within-person changes in MHL over the course of pregnancy. Therefore, future research should employ a longitudinal cohort design to prospectively follow the same group of pregnant women, thereby elucidating the dynamic changes in MHL throughout pregnancy and its long-term effects on maternal and infant outcomes. Finally, this study focused on low-risk pregnancies and excluded women with high-risk conditions, while this approach controlled for confounding by disease severity, it may limit the generalizability of the findings to a broader population of pregnant women. Therefore, future studies may include high-risk pregnant populations and compare their MHL rates with those of low-risk populations to make the results more representative. In addition, future studies could explore sensitivity analyses using alternative cutoffs to examine the stability of the prevalence estimate.

## 5 Conclusions

The MHL among pregnant women was at low rate in Chongqing. The rate of MHL was positively associated with a higher educational level of participants, worries about the effect of pregnancy on work, and attendance at pregnancy school but negatively associated with living in rural areas, ethnic minorities, and abnormal results of prenatal screening. Therefore, healthcare professionals should provide individualized and multifaceted health education approaches for pregnant women with diverse characteristics, with particular attention to those in townships, from ethnic minority backgrounds, or with abnormal prenatal screening results. Strengthening support for these groups may help improve perinatal maternal and infant health outcomes.

## Data Availability

The raw data supporting the conclusions of this article will be made available by the authors, without undue reservation.
