# Supplementary material for: Maternal health literacy status and associated factors among pregnant women: a cross-sectional study in Chongqing, China
Source: Front Public Health. 2026 Jul 16;14:1824347. doi: 10.3389/fpubh.2026.1824347 (PMC13422526; doi:10.3389/fpubh.2026.1824347)
Supplement: Supplementary file 1 [file Data_Sheet_1.docx]

# Maternal Health Literacy Questionnaire

**Instructions for completing the questionnaire:**
Please answer this questionnaire truthfully. There is no need to search for answers online or ask others. If you really do not know the answer, you may choose “I don’t know.” Please read each question carefully while completing the questionnaire.

**1. Advanced maternal age refers to pregnant women older than ______.**

A. 30 years old
B. 32 years old
C. 35 years old
D. 38 years old
E. I don’t know

**2. Folic acid supplementation should begin ______.**

A. 3 months before pregnancy
B. After pregnancy is confirmed
C. 1 month after pregnancy
D. 3 months after pregnancy
E. I don’t know

**3. The pathogens of some diseases can infect the fetus through the placenta. Therefore, corresponding laboratory tests should be performed during the first prenatal examination to detect abnormalities and provide timely treatment. These diseases are ______.**

A. Syphilis, AIDS, and hepatitis B
B. Hypertension, diabetes, and coronary heart disease
C. Schistosomiasis and chronic gastritis
D. I don’t know

**4. Among the prenatal examination items in the third trimester, which of the following does not need to be checked every time?**

A. Urinary protein
B. Fetal heart rate
C. Fundal height
D. Abdominal circumference
E. I don’t know

**5. Early pregnancy reactions generally occur at around ______ weeks of pregnancy.**

A. 6
B. 7
C. 8
D. 9
E. I don’t know

**6. Normal blood pressure in adults is defined as systolic blood pressure below ______ mmHg and diastolic blood pressure below ______ mmHg.**

A. 150/70
B. 150/80
C. 140/90
D. 180/100
E. I don’t know

**7. Toxoplasmosis is most likely associated with contact with which type of pet?**

A. Cat
B. Dog
C. Rabbit
D. Mouse
E. I don’t know

**8. Full-term delivery refers to delivery occurring from ______ completed weeks of gestation to less than 42 weeks.**

A. 36
B. 37
C. 38
D. 40
E. I don’t know

**9. Before delivery, if a pregnant woman experiences which of the following conditions, she may not need to rush to the hospital immediately?**

A. Bloody show
B. Heavy vaginal bleeding with or without abdominal pain
C. Regular uterine contractions with labor pain
D. Rupture of membranes, also known as water breaking
E. I don’t know

**10. Which of the following statements about vaginal delivery and cesarean section is incorrect?**

A. Cesarean section causes less pain, the fetus is not squeezed, and the mother can maintain her body shape
B. Vaginal delivery causes less trauma to the mother and allows faster postpartum recovery
C. Infants delivered by cesarean section have later contact with the mother’s intestinal flora because they do not pass through the birth canal, which may affect the development of their immune system
D. Whether delivered vaginally or by cesarean section, infants should be breastfed, which is beneficial to the development of their immune system
E. I don’t know

**11. Postpartum blues are mainly related to ______.**

A. Introverted personality
B. Irritability
C. Rapid changes in hormone levels after delivery
D. Inadequate care from family members
E. I don’t know

**12. Macrosomia refers to a newborn with a birth weight reaching or exceeding how many grams?**

A. 3800
B. 4000
C. 4200
D. 4500
E. I don’t know

**13. After birth, newborns may experience physiological weight loss due to water loss and insufficient breast milk production. This generally does not exceed ______ of birth weight, and the weight usually recovers in about 7–10 days.**

A. 5%
B. 10%
C. 15%
D. 20%
E. I don’t know

**14. At one month of age, it is normal for a newborn’s weight to increase by more than ______ grams.**

A. 500
B. 600
C. 700
D. 800
E. I don’t know

**15. Physiological jaundice in newborns usually subsides in about ______ days.**

A. 1–3
B. 4–6
C. 7–10
D. 11–14
E. I don’t know

**16. How long after birth does the umbilical cord usually fall off in newborns?**

A. Within 1 week
B. 1–2 weeks
C. 2–3 weeks
D. 3–4 weeks
E. I don’t know

**17. Which of the following is not a normal physiological phenomenon in the neonatal period?**

A. Pseudomenstruation
B. Breast swelling and milk secretion
C. “Mantis mouth” and “Epstein pearls”
D. Thrush
E. I don’t know

**18. The anterior fontanelle of an infant generally closes at ______ months of age.**

A. 3–6
B. 6–12
C. 12–18
D. 18–24
E. I don’t know

**19. The timing of eruption of primary teeth varies greatly among infants. If the first tooth has not erupted by ______ months, the infant should be taken to the hospital for examination.**

A. 12
B. 14
C. 16
D. 18
E. I don’t know

**20. After birth, infants should be vaccinated strictly according to the national immunization schedule. Which vaccine should be administered at birth, at one month, and at six months of age?**

A. BCG vaccine
B. Hepatitis B vaccine
C. DTP vaccine
D. Measles vaccine
E. I don’t know

**21. Breastfeeding has many benefits. Which of the following is incorrect?**

A. Breast milk contains immunologically active substances with anti-infective effects
B. The protein in breast milk is mainly whey protein, which is easy to digest and absorb
C. Oligosaccharides in breast milk can promote the growth of intestinal probiotics
D. The contents of vitamins and minerals in breast milk are higher than those in cow’s milk
E. I don’t know

**22. When exclusive breastfeeding is not possible due to various conditions, the preferred breast milk substitute should be ______. For infants at risk of allergy, ______ should be selected.**

A. Infant formula; partially hydrolyzed infant formula
B. Infant formula; DHA-containing infant formula
C. Infant formula; skimmed milk
D. Infant formula; goat milk
E. I don’t know

**23. For pregnant women with normal pre-pregnancy weight, the recommended appropriate weight gain during pregnancy is ______ kilograms.**

A. 16
B. 14
C. 12
D. 10
E. I don’t know

**24. Which activity is not suitable during pregnancy?**

A. Ball games and mountain climbing
B. Swimming
C. Pregnancy exercises and prenatal yoga
D. Walking
E. I don’t know

**25. During the postpartum confinement period, which of the following practices is inappropriate for new mothers?**

A. Brushing teeth
B. Taking a shower
C. Eating fruit
D. Dieting to lose weight
E. I don’t know

**26. The World Health Organization recommends that after complementary foods are introduced, breastfeeding should still be continued as long as possible, up to ______ months or beyond.**

A. 6
B. 12
C. 18
D. 24
E. I don’t know

**27. It is best to allow the baby to suck the breast within ______ hour(s) after delivery to promote milk secretion, namely early sucking and early initiation of breastfeeding.**

A. 1
B. 2
C. 3
D. 4
E. I don’t know

**28. In addition to receiving vaccinations on time, infants should receive at least ______ comprehensive physical examinations within the first year of life.**

A. 1
B. 2
C. 3
D. 4
E. I don’t know

**29. In the third trimester, it is recommended to increase the daily intake of protein-rich foods, including fish, poultry, eggs, and lean meat, by a total of approximately ______ grams.**

A. 50–100
B. 100–150
C. 150–200
D. 200–250
E. I don’t know

**30. According to the Chinese Dietary Reference Intakes, the recommended calcium intake for women in the second and third trimesters is ______ mg/day and ______ mg/day, respectively.**

A. 800, 1000
B. 1000, 1200
C. 1000, 1000
D. 1200, 1500
E. I don’t know

**31. From which day should the expected date of delivery be calculated?**

A. The day of sexual intercourse when conception may have occurred
B. The day when the urine pregnancy test was positive
C. The first day of the last menstrual period
D. The last day of the last menstrual period
E. I don’t know

**32. Pregnant women usually begin to perceive fetal movement at approximately 18–20 weeks of gestation. The method for counting fetal movements is as follows: count for 1 hour each in the morning, afternoon, and evening, add the three counts together, and multiply by 4 to obtain the 12-hour count. Normally, it should be ______ times or more.**

A. 10
B. 20
C. 30
D. 40
E. I don’t know

**33. When an infant or young child has a fever with a body temperature exceeding ______, appropriate cooling measures, such as physical cooling or antipyretic medication, should be taken promptly under the guidance of a doctor.**

A. 37.5℃
B. 38.0℃
C. 38.5℃
D. 39.0℃
E. I don’t know

**34. In a relatively quiet state, the respiratory rate of infants aged 2 days to 2 months should not exceed ______ breaths/min, and that of infants aged 2 months to 1 year should not exceed ______ breaths/min.**

A. 30, 20
B. 40, 30
C. 50, 40
D. 60, 50
E. I don’t know

**35. Infants require special attention and should be supervised by a caregiver at all times. Which of the following practices is inappropriate?**

A. Installing safety guardrails on balcony windows, stairs, and other places
B. Keeping medicines, detergents, pesticides, and similar items at home out of the infant’s reach
C. Leaving the infant alone in a baby walker
D. Installing protective covers on electrical outlets at home
E. I don’t know
